# Finger Vein Recognition Based on Local Directional Code

**DOI:** 10.3390/s121114937

**Published:** 2012-11-05

**Authors:** Xianjing Meng, Gongping Yang, Yilong Yin, Rongyang Xiao

**Affiliations:** School of Computer Science and Technology, Shandong University, Jinan 250101, China; E-Mails: rongmengyuan@gmail.com (X.M.); gpyang@sdu.edu.cn (G.Y.); ylyin@sdu.edu.cn (Y.Y.); canyueyang@126.com (R.X.)

**Keywords:** finger vein recognition, local directional code, local line binary pattern, local gradient orientation, webber local descriptor

## Abstract

Finger vein patterns are considered as one of the most promising biometric authentication methods for its security and convenience. Most of the current available finger vein recognition methods utilize features from a segmented blood vessel network. As an improperly segmented network may degrade the recognition accuracy, binary pattern based methods are proposed, such as Local Binary Pattern (LBP), Local Derivative Pattern (LDP) and Local Line Binary Pattern (LLBP). However, the rich directional information hidden in the finger vein pattern has not been fully exploited by the existing local patterns. Inspired by the Webber Local Descriptor (WLD), this paper represents a new direction based local descriptor called Local Directional Code (LDC) and applies it to finger vein recognition. In LDC, the local gradient orientation information is coded as an octonary decimal number. Experimental results show that the proposed method using LDC achieves better performance than methods using LLBP.

## 1. Introduction

Recently, there has been much interest in biometric authentication for security purposes. Biometrics or biometric authentication [1,2] refers to automated methods of recognizing a person using behavioral or physiological features, such as, faces [3], irises [4], gaits [5], fingerprints [6,7], veins, *etc*. Biometric features are unique characteristics to an individual which is convenient and more secure than traditional authentication methods. For example, biometric recognition is more reliable than token-based verification methods (keys or ID cards) and knowledge-based methods (passwords or PINs) while attaining higher efficiency and offering a better user experience.

Personal verification based on biometric technology is widely used in door access control, security systems and forensics. However, under special circumstances, these biometric approaches may suffer from some practical limitations. For example, face recognition is susceptible to illumination changes and rotations. Fingerprints [8] are vulnerable to forgery because fingerprints are easily exposed to others. Moreover, the conditions of a finger such as dryness or sweat can also prevent a clear pattern from being obtained.

Due to the limitations mentioned above, new approaches have been proposed to overcome the existing problems. Meanwhile, many new biometric features have been exploited. Finger vein recognition is just such a kind of biometric approach to overcome some of the limitations. In [9], the authors prove that each finger has a unique vein pattern that can be used for personal verification. In addition, finger vein recognition also has lots of advantages over other biometric authentication techniques [10,11]: (1) non-contact; (2) live-body identification; (3) high security; (4) small device size; (5) with ten fingers per person available, if something unexpected happens, other fingers can also be authenticated. The finger vein recognition is widely considered one of the most promising identification technologies in the future [12].

Finger vein recognition includes four main steps: image capturing, pre-processing, feature extraction and matching. The extracted features sometimes seriously affect the performance of the recognition systems. In order to extract better features from the segmented blood vessel network, in [13] the authors extract the finger vein pattern from the unclear image with line tracking, which starts from various positions. In [14], the minutiae features, including bifurcation points and ending points which can be used for geometric representation of the vein patterns' shape are extracted from these vein patterns. In [15], the authors propose a mean curvature method, which regards the vein image as a geometric shape and finds the valley-like structures with negative mean curvatures; an equal error rate of 0.25% is reported with test images from 125 fingers. As mentioned above, most of the currently available finger vein-based recognition methods utilize features from a segmented blood vessel network. However, due to the optical blurring and scattering problems, the finger vein images are not always clear and show irregular shadings [16,17]. Improperly segmented networks may degrade the recognition accuracy dramatically. To solve this problem, binary pattern based methods are proposed and then LBP [18], LDP [19] are applied to the finger vein recognition. A number of LBP variants have also been proposed. For example, Local Ternary Pattern (LTP) [20] uses a three-value encoding instead of two-value encoding as in the original LBP while Local Quinary Pattern (LQP) [21] uses a five-value encoding. In [22], the authors proposed a new LBP variant called Directional Binary Code (DBC) and applied it to near-infrared face recognition. In [23], the authors proposed the use of a Personalized Best Bit Map (PBBM), which is rooted in a local binary pattern (LBP) and the experiments demonstrate that this feature achieves not only better performance, but also high robustness and reliability. Recently, Petpon and Srisuk [24] proposed a new variant of LBP called Local Line Binary Pattern (LLBP) and Rosdi *et al.* [25] applied it to finger vein recognition and the authors demonstrate a better accuracy than both LBP and LDP.

Though finger vein recognition methods can achieve high accuracy using local patterns, it is essentially a kind of network which is hard to extract. As a kind of networks, finger veins contain rich directional information. However, the above mentioned local pattern-based methods have not made full use of the directional information hidden in the finger vein images. Inspired by the Webber Local Descriptor (WLD) [26], we propose a more descriptive local descriptor called Local Directional Code (LDC). LDC is a square local descriptor which needs only four neighbors when encoding, while up to 2(*N*-1) squared neighbors are used for encoding with LLBP, so the computing complexity of LDC is much lower than LLBP. In LDC, we code the gradient orientation information as an octonary decimal number, compared with the LLBP feature which is obtained in both the vertical and horizontal direction, LDC can better reflect the direction information and local features. Our experiments demonstrate that the LDC feature has improved recognition accuracy.

The rest of the paper is organized as follows: Section 2 presents the proposed local directional code (LDC) in detail. Multi-direction analysis is also in this part. Section 3, the proposed method for finger vein recognition is described. Section 4 presents the experiments and results. Finally, Section 5 concludes the paper.

## 2. LDC Image Descriptor

In this section, we describe the proposed local directional code (LDC) in detail. Subsequently, we present how to convert a finger vein image into a LDC image to better explain its expressive ability. In addition, we develop its multi-direction analysis.

### 2.1. LDC

**Figure 1.**
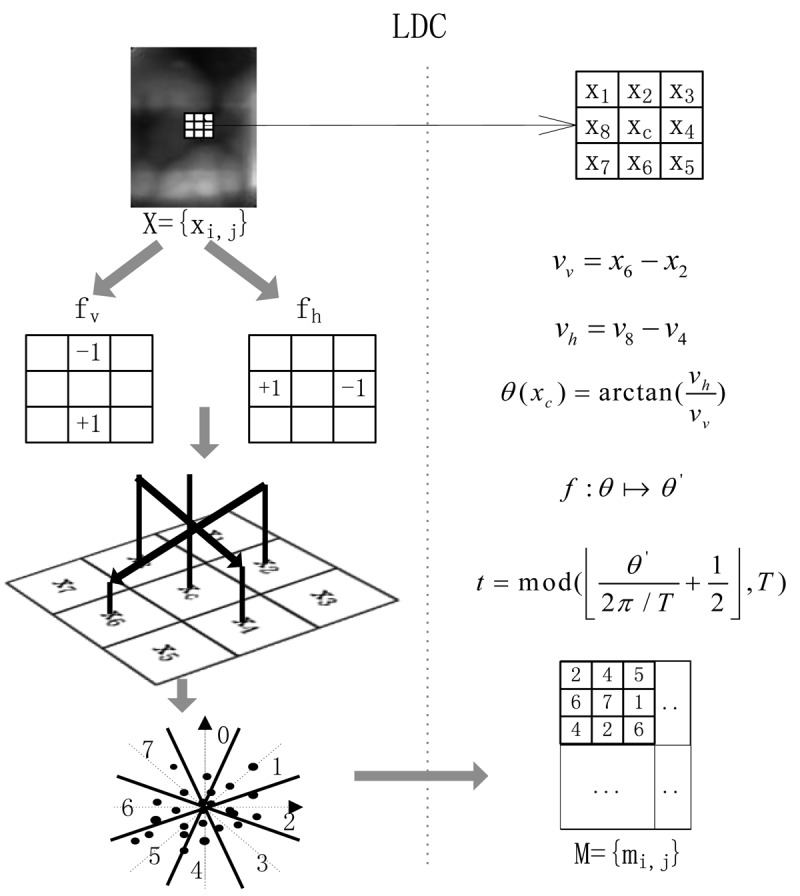
Description of the calculation of the LDC descriptor.

LDC is a kind of local descriptor combined with the gradient direction information inspired by the Webber Local Descriptor (WLD). In WLD, the gradient orientation information, which is used as in [27], is coded as indexes for the differential excitation of each pixel. In our proposed LDC feature, we code the orientation information as a decimal number *t*, as shown in Figure 1.

We use the difference of the neighbors of a pixel as the two components *v_v_* and *v_h_* of the local direction which are the outputs of the vertical filter *f_v_* and horizontal filter *f_h_*:

(1) $$v_{v}=x_{6}-x_{2},v_{h}=x_{8}-x_{4}$$

The gradient orientation is computed as:

(2) $$\theta (x_{c})=\arctan(\frac{v_{h}}{v_{v}})$$

For simplicity, *θ* is further quantized into *T* dominant orientations. Before the quantization, we perform the mapping: *f* : *θ* → *θ′*:

(3) $$\theta^{'}=\arctan2(v_{h},v_{v})+\pi ,\text{and}\arctan2(v_{h},v_{v})=\{\begin{array}{c} \begin{array}{cc} \theta & v_{h}>0,v_{v}>0\\ \pi +\theta & v_{h}>0,v_{v}<0 \end{array}\\ \begin{array}{cc} \theta -\pi & v_{h}<0,v_{v}<0\\ \theta & v_{h}<0,v_{v}>0 \end{array} \end{array}$$

where $\theta \in [-\frac{\pi }{2},\frac{\pi }{2}]$ and *θ′* ∈ [0, 2*π*]. This mapping considers the value of *θ*, computed using (2), and the sign of *v_v_* and *v_h_*. The code function is then as follows:

(4) $$t=\mathrm{mod}(\left\lfloor \frac{\theta^{'}}{2\pi /T}\right\rfloor +\frac{1}{2},T)$$

For example, if *T* = 8, as shown in Figure 1, then *t* ∈ {0, …, 7}. Different value of *t* represents different dominant orientation $\Phi_{t}=\frac{t\pi }{4},$ (*t* = 1, …, *T* − 1). We set *T* = 8 in our finger vein experiments.

In LDC, we code the orientation information into *T* dominant orientations, compared with the LLBP feature which is obtained in both the vertical and horizontal direction, LDC can better reflect the direction information and local features. The gradient orientation contains information from gradient differences of each pixel and their quantitative relations. We then quantize the gradient orientation into *T* dominant directions after a simple mapping. Each value of *T* reflect different trend of intensity, *i.e.*, the trend of finger vein networks. Experiment 3 shows the effects of the parameter *T*.

### 2.2. LDC on Finger Vein Image

As shown in Figure 1, a finger vein image is converted into a code map with the same size. Each element is a directional code ranging from 0 to 7. To convert an image, one pass on the image is enough, which implies that LDC has great efficiency. In order to ensure simplicity, during the computation of the LDC conversion process, the edge pixels of the image are ignored, which implies the dimension of the extracted LDCs is (*W* − 2) × (*H* − 2). The pseudo code of the LDC feature extraction process on a finger vein image is summarized in Algorithm 1. In the algorithm, first, we calculate the two components of the local direction, then the gradient orientation is computed using Equation (2). At last, the gradient direction is further quantized into *T* dominant orientation after a simple mapping using Equation (3). Parameters *W* and *H* refer to the width and height of a finger vein image. The calculation is performed in a 3 × 3 neighborhood.

**Figure 2.**
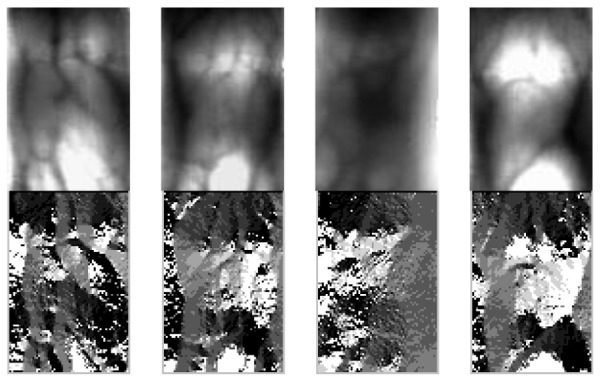
Finger vein images processed by LDC. The first row shows the original finger vein images, the second row shows the LDC images, respectively.

The finger vein images processed using LDC are shown in Figure 2. It should be noted that for viewing convenience, the local directional codes computed for every pixel are normalized to values from 0 to 255. From Figure 2, we can see the discriminablity between different finger vein images.

### 2.3. Multi-direction Analysis

**Figure 3.**
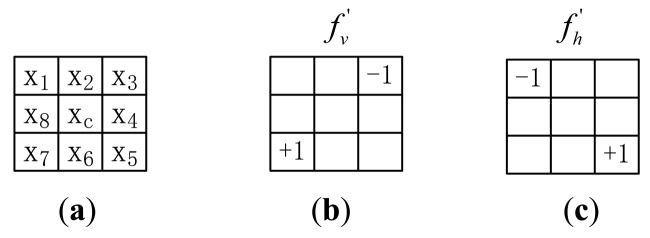
Illustration of 45°-direction analysis. (**a**) 3-square neighbor. (**b**) Vertical filter of LDC in 45° rotation. (**c**) Horizontal filter of LDC in 45° rotation.

The LDC features described above are extracted from the 3 × 3 neighborhood in a vertically crossed way, where the gradient orientation is computed using the outputs of the vertical filter *f_v_* and horizontal filter *f_h_*. We mark it as LDC-00 where 00 means the original LDC without rotation. For the orientations of the finger vein vessel network may vary, we generalize LDC in different directions. In a 3 × 3 neighborhood, the LDC in 45° rotation is given, which is illustrated in Figure 3.

We marked the LDC in 45° rotation as LDC-45. In LDC-45, the difference of the neighbors of a pixel *v_v_*′ and *v_h_*′ are the outputs of the vertical filter *f_v_*′ and horizontal filter *f_h_*′:

(5) $$v_{v}^{'}=x_{7}-x_{3},v_{h}^{'}=x_{5}-x_{1}$$

The gradient orientation of LDC-45 is computed as:

(6) $$\theta (x_{c})=\arctan(\frac{v_{h}^{'}}{v_{v}^{'}})$$

Since we have extended the LDC in multiple directions, we also compare its performance with the original LDC (LDC-00) in Section 4.

## 3. The Proposed Method

**Figure 4.**
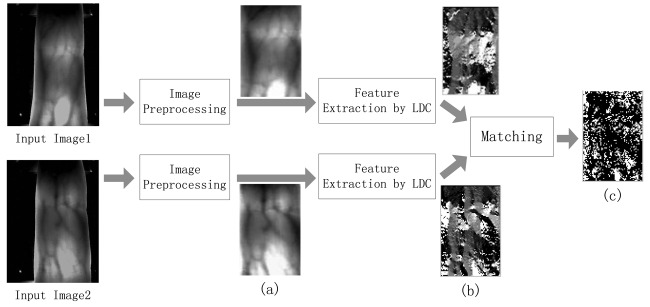
Flowchart of the finger vein recognition method. (**a**) Figure vein image after preprocessing; (**b**) Vision image for LDCs; (**c**) Matching results, here the black region implies the unmatched area, as Image 1 and Image 2 are from different classes, only 27 percent of the LDCs are matched.

In this section, we describe the proposed method for finger vein recognition. The vein recognition procedure includes preprocessing, feature extraction using Local Directional Code (LDC) and the computation of matching scores. Feature extraction was already described in Section 2. Figure 4 shows the block diagram of the proposed finger vein recognition method.

### 3.1. Preprocessing

There are four main steps in the image preprocessing operation, which are image gray processing, ROI extraction, size normalization and gray normalization.

#### Image Gray Processing

**Figure 5.**
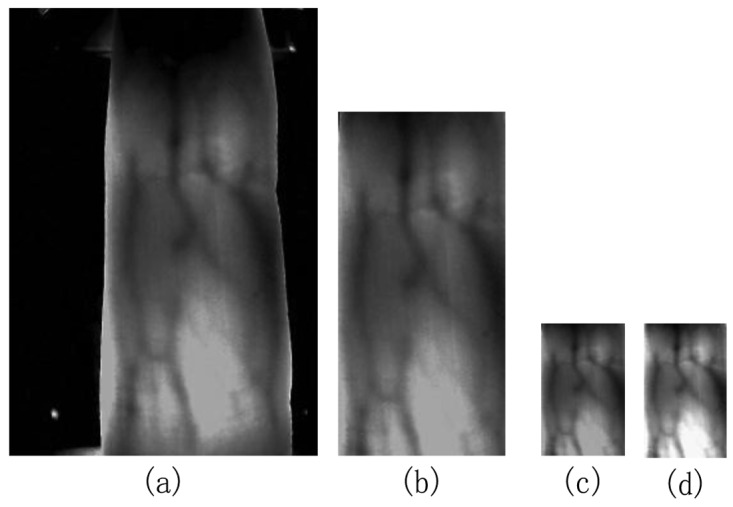
Examples of preprocessing. (**a**) Original finger vein image; (**b**) ROI of (a); (**c**) Finger vein image after size normalization; (**d**) Finger vein image after gray normalization.

The finger vein image captured by the device in this paper, which is shown in Figure 5(a), is a 24-bit color image with a size of 320 × 240. In order to reduce the computational complexity, the original image is transformed into an 8-bit gray image based on the RGB to Grayscale Equation:

(7) Y=R×0.299+G×0.587+B×0.114

where R, G and B denote the decimal values of the red, green and blue color components.

#### ROI Extraction

As the acquired finger vein image has an unwanted black background which may interfere with the recognition process, we employ an edge detection method to segment the finger vein region, which is defined as the Region of Interest (ROI), from the grayscale image. A Sobel operator with a 3 × 3 mask $\left|\begin{array}{ccc} -1 & 0 & 1\\ -2 & 0 & 2\\ -1 & 0 & 1 \end{array}\right|$ is used for detecting the edges of a finger. The width of the finger region can be obtained based on the maximum and minimum abscissa values of the finger profile and the height of the finger region can be similarly detected. A rectangle region, *i.e.*, the ROI, can be captured based on the two values.

#### Size Normalization

The size of the ROI is different from image to image due to personal factors such as different finger size and changing location. Therefore it is necessary to normalize the ROI region to the same size before feature extraction. We use the bilinear interpolation for size normalization in this paper, and the size of the normalized ROI is set to be 96 × 64.

#### Gray Normalization

In order to extract efficient features, gray normalization is used to obtain a uniform gray distribution. In this paper, the normalized image *X* is defined as follows:

(8) $$X(i,j)=\frac{X^{'}(i,j)-X_{\min}}{X_{\max}-X_{\min}}\times 255$$

(9) $$X_{\min}=\underset{i\in [1,H],j\in [1,W]}{\min}X^{\prime }(i,j)$$

(10) $$X_{\max}=\underset{i\in \left[1,H\right],j\in \left[1,W\right]}{\max}X^{'}(i,j)$$

where *X*(*i,j*) is the gray value of pixel at position (*i,j*) of the original image, *X*_min_ denotes the minimum gray value of the original image and *X*_max_ denotes the maximum gray value of the original image.

### 3.2. Matching

The proposed method estimates the similarities between the extracted local directional codes (LDC) and the enrolled codes of a certain individual. The matching score is measured using the following formula:

(11) $$\text{Score}=\frac{\sum_{i}\sum_{j}Mt(i,j)}{\text{CodeNum}}$$

(12) $$Mt(i,j)=\{\begin{array}{cc} 1 & \begin{array}{cc} \text{if} & \text{codeA}(i,j)=\text{codeB}(i,j) \end{array}\\ 0 & \text{other} \end{array}$$

In Equation (9), *Mt* [as shown in Figure 4(c)] is a bitmap used to signify whether *codeA*(*i,j*) and *codeB*(*i,j*) have the same LDC value. *CodeNum* is the total number of LDCs of the matching area, to be more specific, the number of elements in *Mt*. Apparently, the *Score* ranges from 0 to 1.

## 4. Experimental Results

### 4.1. The Experimental Database

The experiments were conducted using our finger vein database which was collected from 34 individuals (20 males and 14 females, Asian race) who are students, professors and staff at our school. To acquire these natural finger vein images, each individual participated in two sessions, separated by 20 days. The age of the participants was between 19 and 48 years. Each individual provides 4 fingers, which are left index, left middle, right index and right middle fingers, each of which contributes 30 images. Consequently, our finger vein database includes 4,080 (34 × 4 × 30) finger vein images. The finger vein image captured by the device used in this paper, which is shown in Figure 5, is a 24-bit color image with a size of 320 × 240. The original spatial resolution of the data is 320 × 240, After ROI extraction and Size Normalization, the size of the region used for feature extraction is reduced to 96 × 64. Samples collected from the same finger belong to the same class. Therefore, there are 136 classes, where each class contains 30 samples in our database.

### 4.2. The Experiment Settings

All the experiments are implemented in MATLAB, and performed on a PC with a 2.4 GHz CPU and 2.0 G memory. In the experiment, the LDC is extracted from three square neighbors in both 0 and 45 degrees, which are labeled LDC-00 and LDC-45. The experiments are designed to multilaterally evaluate the proposed method: (1) Experiment 1 evaluates the performance of the proposed method in verification mode, and compared with the LLBP based method. (2) In Experiment 2 we compare the proposed method and LLBP in the identification mode. (3) Experiment 3 discusses the influence of the parameter setting of *T*. (4) Experiment 4 measures the average processing time.

### 4.3. Experiment 1

We performed Experiment 1 on the established database in the verification mode. In this mode, the class of the input finger vein (test sample) is known, and each sample is matched with all the other samples from the same class and first six samples of the other 135 classes. A successful matching is called intraclass matching or genuine, if the two matching samples are from the same class. Otherwise, the unsuccessful matching is called interclass matching or imposter. As mentioned above, we use full matching in intraclass matching (each sample is matched with all the other samples from the same class), and each sample is matched with the first six samples from the other 135 classes. Consequently, there are 59,160 $\left(136\times C_{30}^{2}\right)$ intraclass matching and 330,480 (136 × 6 × 135 × 6) interclass matching in total. In this paper, the performance of a system is evaluated by the EER (equal error rate), the FRR (false rejection rate) at zero FAR(false accept rate), and the FAR at zero FRR. The EER is the error rate when the FRR equals the FAR and therefore suited for measuring the performance of biometrics systems because the FRR and FAR are treated equally. On the other hand the FRR at zero FAR is suited for high security systems, as in those systems, false acceptance errors are much more critical than false rejection errors. On the contrary, the FAR at zero FRR shows the acceptance rate of impostors when more genuine rejected is desired.

**Figure 6.**
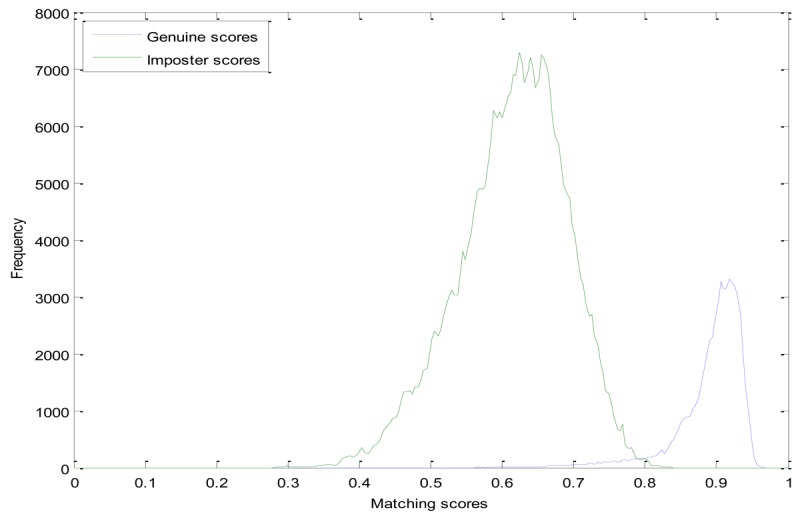
Matching score distribution of the LLBP-based method.

**Figure 7.**
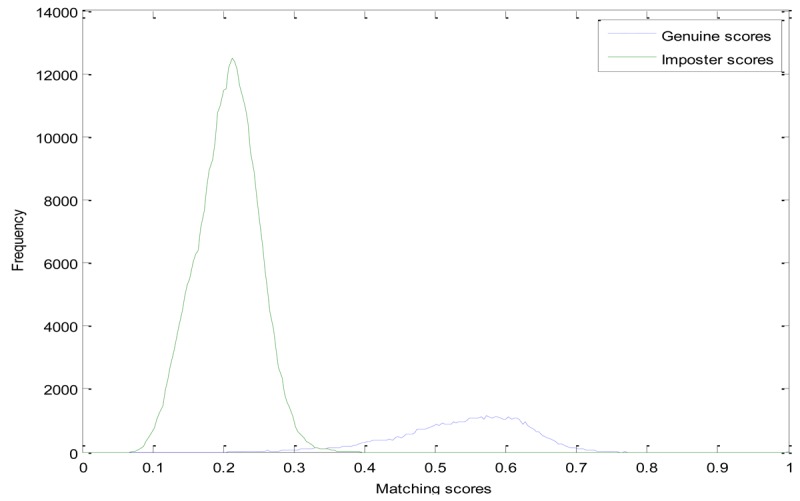
Matching score distribution of the LDC -00-based finger vein recognition.

**Figure 8.**
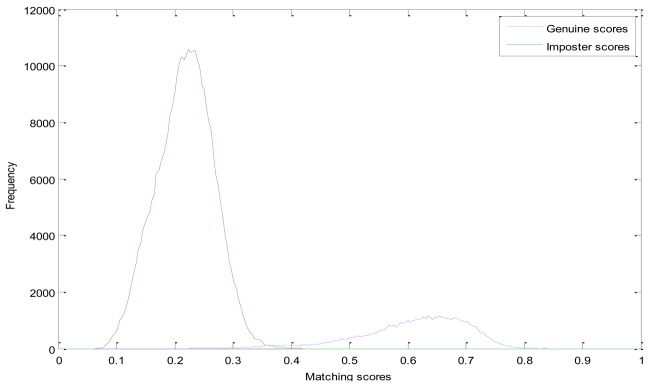
Matching score distribution of the LDC-45-based finger vein recognition.

In the experiments, we compare the proposed method with the LLBP based method. Genuine and Imposter matching score distributions of these two methods are shown in Figures 6 and 7, respectively. From Figure 6, we can see that, the genuine matching score and the imposter matching score of the LLBP based method are overlapped between 0.67 and 0.83, while the genuine and imposter matching score of the LDC-00-based method is mainly between 0.35 and 0.7, 0.08 and 0.35, and of the LDC-45-based method mainly between 0.4 and 0.8, and the imposter matching score is mainly between 0.08 and 0.38, as we can see from Figures 7 and 8.

**Figure 9.**
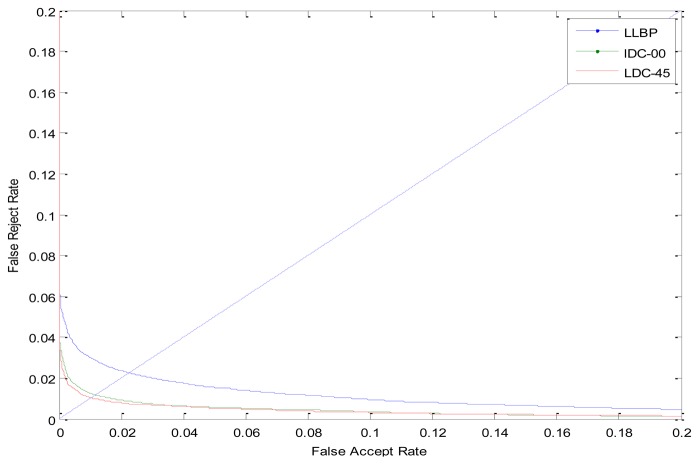
The ROC curve in the verification mode.

The ROC curves are shown in Figure 9. The FRR at zero FAR and FAR at zero FRR values are listed in Table 1. From Figure 9 and Table 1, we can see that the proposed LDC method achieves a much lower EER than the LLBP-based method. This indicates that the LDC feature can better reflect the characteristics of a certain individual. Besides, it can make a better use of the directional information which can better illustrate the difference between the individuals.

### 4.4. Experiment 2

In Experiment 2, we compare the proposed method and LLBP in the identification mode. Closed-set identification experiments (identification is performed only for individuals who are present in the enrollment database) were also conducted. In the identification mode, we don't know the class to which the input finger vein images belong to and have to verify it. In this mode, we use the first 10 finger vein images of each class as test samples and randomly select one finger vein from the remaining 20 samples of each class as templates. Therefore, there are 136 templates and 1,360 (130 × 10) probes in total. The probes were matched with all the template models. For each probe, we can get its rank according the matching results using just one pass:

(13) $$\text{ran}k_{j}=1+\sum_{i\in [1,136]}\left[\text{list}(i)>\mathrm{list}(j)\right]$$

**Figure 10.**
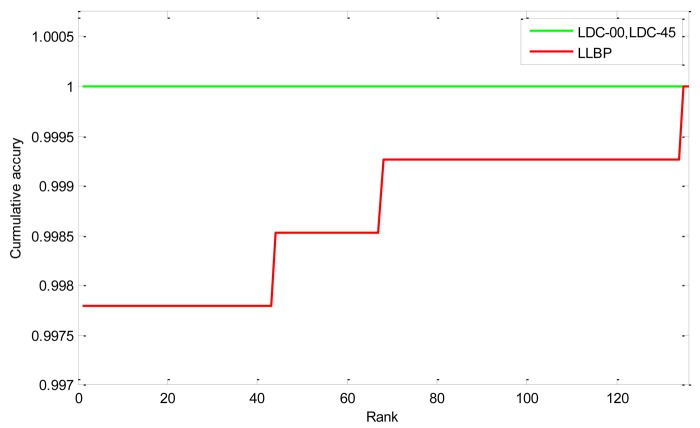
Cumulative match curves by different methods.

Here, *rank_j_* implies that the probe belong to class *j*. *list* represents the vector of the matching scores. [*list*(*i*) > *list*(*j*)] returns 1 if [*list*(*i*) > *list*(*j*)] is true. Then we can get the cumulative match curves as shown in Figure 10. The rank one recognition rate and the lowest rank of perfect recognition (*i.e.*, the lowest rank when the recognition rate reaches 100%) are listed in Table 2. From the experimental results we can see that the proposed method is much better than that of the LLBP based method. The rank one rates of the proposed method LDC-00 and LDC-45 are both 100%, indicating that all the probes are identified correctly.

### 4.5. Experiment 3

**Figure 11.**
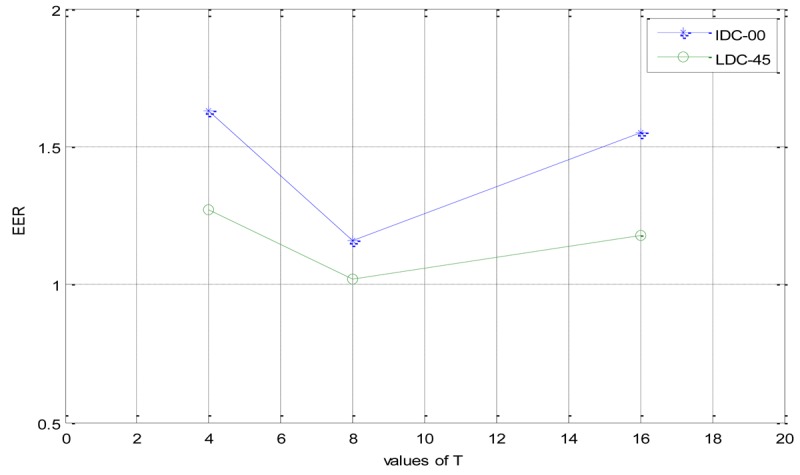
The effects of using different vaules of parameter *T*.

In this section, we discuss the influence of the parameter setting of *T*. Like WLD [26], the setting of *T* is still a trade-off between discriminability, statistical reliability and tolerance to noises. In general, if the parameter *T* becomes larger, the number of different dominant orientation becomes larger, and thus the LDCs become more discriminable. However, the number of pixels in each dominant orientation becomes smaller because the size of the input image is fixed. This degrades the statistical reliability of the LDCs. If the number of pixels belonging to each dominant orientation becomes too small, it will in turn degrade the discriminability of the LDCs because of its poor statistical reliability. Larger numbers of dominant orientations could cause a poor tolerance to noises. In contrast, if parameter *T* becomes smaller, the pixels in each dominant orientation becomes larger and acquire a better tolerance to noise, the LDCs become statistically more reliable. However, if *T* is too small, the number of the dominant orientations also becomes too small and degrades the discriminability of the LDCs. The experimental results when varying the parameter *T* are given is Figure 11.

From Figure 11, we can see that, when the parameter *T* is equal to 8, the system can achieve a comparatively best tradeoff between discriminability and statistical reliability.

### 4.6. Experiment 4

In Experiment 4, the average processing times were measured, as shown in Table 3. The experiment is implemented in MATLAB, and conducted on a PC with a 2.4 GHz CPU and 2.0 G memory. The average feature extraction time of LDC is 16 ms per image, and the average matching time between two LDCs is 12 ms. The preprocessing time is 53 ms per image. From Table 3 we can see that the total running time for one match of LDC is slightly faster than LLBP. In a word, the proposed method can be used in real-time applications.

## 5. Conclusions and Future Work

Extraction of robust features from finger vein images is an important issue in finger vein-based biometric authentication. In this paper, we represent a novel feature extraction method based on the directional information and apply it to finger vein recognition. Though finger vein recognition methods can achieve high accuracy using local patterns, it is essentially a kind of inexact extractable blood vessel network. As a kind of network, finger veins contain a wealth of directional information. In the experimental results, we can see that LDC is more suitable for finger vein recognition than the LLBP- based method. In the future, we plan to design a new feature extraction method considering both the direction and global texture of the finger vein networks.

## Figures and Tables

**Table 1.** Verification performance by different methods.

**Method**	**EER**	**FAR at zero FRR**	**FRR at zero FAR**
LLBP	0.0225	0.7621	0.0877
LDC-00	0.0116	0.4962	0.0653
LDC-45	0.0102	0.4514	0.0468

**Table 2.** Identification performance by different methods.

**Method**	**Rank one Recognition Rate**	**Lowest Rank of Perfect Recognition**
LLBP	99.78%	135
LDC-00	100%	1
LDC-45	100%	1

**Table 3.** The average preprocessing time for the LDC method.

**Method**	**Preprocessing**	**Feature Extraction**	**Matching**
LDC	53 ms	16 ms	12 ms
LLBP	53 ms	22 ms	8 ms

**Algorithm 1.** The LDC feature extraction.

**Require**: Image *X* = {*x_i,j_*∣*i* ∈ (1, …*H*), *j* ∈ (1, …*W*)}	
**Ensure**: LDC feature map *M* = { *m_i,j_*∣*i* ∈ (1, …*H* − 2), *j* ∈ (1, …*W* − 2)}	
1.	Allocate 2D array *M*, where *M*[*i*, *j*] is intended to hold the local directional code of pixel at position (*i* − 1, *j* −1);
2.	for *i* = 2 to *H* − 1
3.	for *j* = 2 to *W* − 1
4.	get *v_v_* = *x_i, j_*_+1_ − *x_i, j_*_−1_ and *v_h_* = *x_i_*_−1_*_, j_* − *x_i_*_+1_*_, j_*; // Calculate the two components of the local direction.
5.	calculate *θ*(*x_i, j_*) using formula(2), $\theta (x_{i,j})=\arctan(\frac{v_{h}}{v_{v}})$; // Compute the gradient orientation.
6.	If *v_h_* > 0 and *v_v_* > 0 then // Mapping *θ* to *θ*′.
7.	*θ*′ = *θ* + *π*;
8.	else if *v_h_* > 0 and *v_v_* < 0 then
9.	*θ*′ = *θ* + 2*π*;
10.	else if *v_h_* < 0 and *v_v_* < 0 then
11.	*θ*′ = *θ*;
12.	else if *v_h_* < 0 and *v_v_* > 0 then
13.	*θ*′ = *θ* + *π*
14.	end if;
15.	calculate *M*[*i* − 1, *j* − 1] using formula (4), $M[i-1,j-1]=\mathrm{mod}(\left\lfloor \frac{\theta^{'}}{2\pi /T}\right\rfloor +\frac{1}{2},T)$; // Further quantize *θ* into *T* dominant directions.
16.	end for;
17.	end for;
18.	Return *M*.
